# Advances in Tissue Engineering and Regenerative Medicine: Biomaterials, Biofabrication, Cell-Based and Cell-Free Therapies, and Applications in Reconstructive and Aesthetic Medicine

**DOI:** 10.3390/cells15171518

**Published:** 2026-08-24

**Authors:** Caijun Jin, Zhiyuan Ding, Huizhen Ming, JungHee Shim, Vo Tien Huy, Pham Ngoc Chien, Kyung Min Choi, Chan Yeong Heo

**Affiliations:** 1 Department of Plastic and Reconstructive Surgery, College of Medicine, Seoul National University, Seoul 03080, Republic of Korea; jcjking96@snu.ac.kr (C.J.); jiwon4@snu.ac.kr (Z.D.); ming921@snu.ac.kr (H.M.); lionheo@snu.ac.kr (C.Y.H.); 2 Department of Plastic and Reconstructive Surgery, Seoul National University Bundang Hospital, Seongnam 13620, Republic of Korea; xmylife@empal.com; 3 Department of Anatomy, VNU University of Medicine and Pharmacy, Vietnam National University, Hanoi 100000, Vietnam; dr.richardhuy@gmail.com; 4 Department of Chemical and Biological Engineering, Sookmyung Women’s University, Seoul 04310, Republic of Korea; 5 LabInCube Co. Ltd., Cheongju 28116, Republic of Korea

**Keywords:** tissue engineering, regenerative medicine, biomaterials, 3D bioprinting, extracellular vesicles, stem cells, biomodulators, skinboosters, extracellular matrix, skin regeneration

## Abstract

Tissue engineering and regenerative medicine are shifting from passive tissue replacement toward instructive platforms that regulate cellular behavior, immune responses, vascularization, and extracellular matrix remodeling. This review examines recent advances in natural, synthetic, composite, and stimuli-responsive biomaterials, biofabrication and 3D bioprinting, stem and progenitor cell therapies, extracellular vesicles and other cell-free products, immunomodulatory scaffolds, skin organoids and organ-on-a-chip systems, nanotechnology, and artificial intelligence-assisted design. Particular emphasis is placed on plastic, reconstructive, and aesthetic applications, including skin and wound repair, craniofacial bone and cartilage regeneration, peripheral nerve reconstruction, vascularization, and dental and periodontal repair. The review also considers biomodulators and skinboosters as emerging regenerative-aesthetic interventions that aim to improve dermal hydration, fibroblast activity, collagen remodeling, and skin quality rather than provide volume replacement alone. Importantly, these technologies differ substantially in translational maturity, ranging from in vitro and preclinical platforms to early clinical interventions, established clinical products, and commercially available treatments for which durable regenerative efficacy remains incompletely validated. Throughout this review, biological plausibility and preclinical efficacy are therefore distinguished from human clinical evidence, regulatory or established clinical use, and commercial availability. Progress will require standardized characterization, mechanism-linked potency assays, clinically relevant models, and outcome measures that capture functional integration, durability, safety, and aesthetic performance.

## 1. Introduction

Tissue engineering and regenerative medicine seek to restore damaged tissues by integrating cell biology, biomaterials science, bioengineering, and clinical medicine [1]. Conventional treatments such as pharmacotherapy, prosthetic implantation, reconstructive surgery, and transplantation can improve function, but often do not fully re-establish native tissue architecture, biological activity, or long-term homeostasis [2].

The classical tissue-engineering framework combines cells, scaffolds, and bioactive molecules [3]. Cells provide regenerative capacity, scaffolds offer three-dimensional structural support, and molecular cues regulate proliferation, migration, differentiation, angiogenesis, and extracellular matrix remodeling [4]. This framework has expanded to include instructive biomaterials, 3D bioprinting, extracellular vesicles, organoids, gene-delivery systems, immunomodulatory scaffolds, nanotechnology, and artificial intelligence-assisted design [5]. The field has therefore moved beyond structural replacement toward active control of the regenerative microenvironment.

Successful regeneration depends on coordinated interactions among resident cells, recruited progenitors, immune and endothelial cells, nerves, extracellular matrix, and biochemical signals [6]. Accordingly, current strategies aim not only to fill defects or replace lost cells, but also to regulate inflammation, promote vascularization, guide matrix remodeling, and restore tissue-specific function [7]. Cell-based therapies, particularly those using mesenchymal stromal cells and induced pluripotent stem cells, remain important, although their translation is constrained by limited survival, donor variability, senescence, immune compatibility, tumorigenic risk, manufacturing complexity, and regulatory burden [8]. These limitations have accelerated interest in cell-free approaches, including secretomes, extracellular vesicles, growth-factor delivery, and nucleic-acid-based systems.

Skin represents a particularly relevant target because it is accessible, clinically measurable, and affected by wounds, scars, aging, photodamage, inflammation, and extracellular matrix degradation [9]. Regenerative aesthetics has consequently expanded from passive volume correction toward interventions intended to modulate skin quality and extracellular-matrix remodeling [10]. Hyaluronic acid-based skin-quality injectables, polynucleotides, polydeoxyribonucleotide, calcium hydroxyapatite, poly-L-lactic acid, polycaprolactone, peptides, and related formulations have entered aesthetic practice to varying degrees. However, their regulatory status, mechanistic evidence, and clinical support for durable regenerative effects differ substantially among products and jurisdictions.

**Figure 1:**
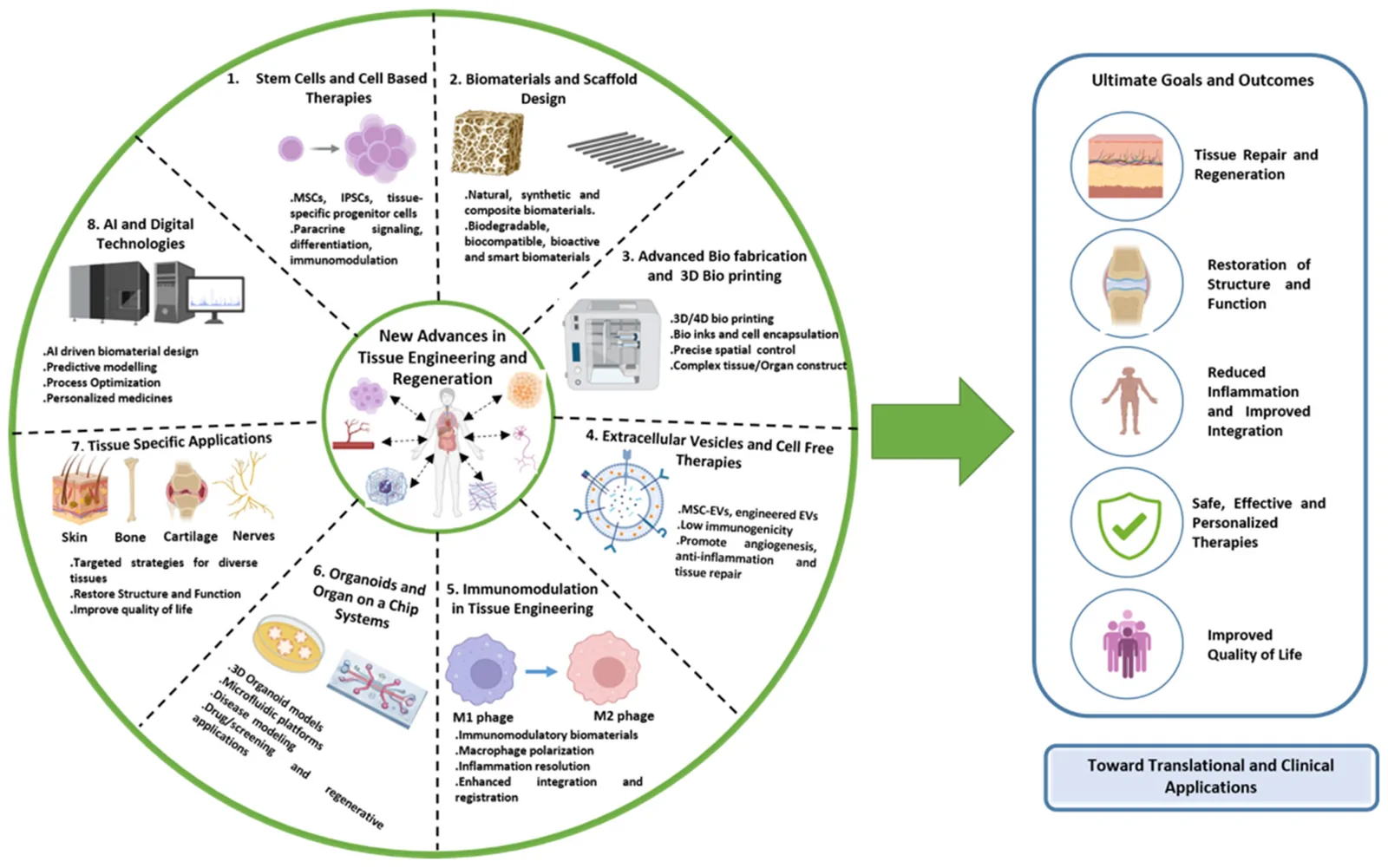
Overview of new advances in tissue engineering and regeneration. Conceptual overview of major advances in tissue engineering and regenerative medicine and their convergence toward tissue repair, functional restoration, improved host integration, personalized therapy, and clinical translation. The figure was created in Microsoft PowerPoint using graphical elements obtained from BioRender.com.

Despite rapid progress, clinical translation remains limited by inadequate vascularization and innervation, fibrosis, poor host integration, insufficient mechanical performance, batch variability, manufacturing cost, and limited long-term evidence [11]. This review therefore examines recent advances in biomaterials, biofabrication, cell-based and cell-free therapies, immunomodulation, organoids, nanotechnology, artificial intelligence, and tissue-specific applications through the lens of plastic, reconstructive, and aesthetic medicine. Across these areas, particular attention is given to clinically relevant problems including wound and scar repair, vascularized soft-tissue reconstruction, durable volume and contour restoration, craniofacial bone and cartilage defects, peripheral nerve recovery, and regenerative skin-quality improvement. As summarized in Figure 1, these approaches represent complementary components of a regenerative framework in which biological, material, and biofabrication strategies converge toward tissue repair, functional restoration, improved host integration, and clinically meaningful reconstructive and aesthetic outcomes.

## 2. Review Methodology

This narrative review was developed through structured literature searches of PubMed/MEDLINE, Web of Science, and Scopus, with the literature updated through July 2026. Search concepts combined terms related to tissue engineering and regenerative medicine with biomaterials, scaffolds, biofabrication, 3D bioprinting, mesenchymal stromal cells, induced pluripotent stem cells, extracellular vesicles, immunomodulation, organoids, organ-on-a-chip systems, nanotechnology, artificial intelligence, wound healing, skin regeneration, scar modulation, soft-tissue reconstruction, biomodulators, skinboosters, calcium hydroxyapatite, poly-L-lactic acid, polycaprolactone, polynucleotides, and polydeoxyribonucleotide. Priority was given to peer-reviewed literature published during the past decade, while earlier landmark studies were retained when necessary to establish key concepts or clinical context.

Original studies, clinical investigations, systematic reviews, consensus papers, and mechanistically relevant reviews were considered when they addressed biological mechanisms, material performance, regenerative efficacy, safety, manufacturing, or clinical translation. Non-peer-reviewed promotional materials and claims that could not be supported by scientific literature were not used as evidence of therapeutic efficacy. Because this article is a narrative rather than systematic review, formal meta-analysis and risk-of-bias scoring were not performed. To improve the interpretation of translational maturity, the evidence discussed in this review was qualitatively categorized according to the stage at which a technology had been evaluated: in vitro or ex vivo studies, small-animal studies, large-animal studies, early human clinical investigations, established or regulator-authorized clinical use, and commercial availability with incomplete clinical or mechanistic validation. These categories were not intended as a formal hierarchy of evidence or risk-of-bias assessment, but rather as a framework for distinguishing experimental promise from demonstrated clinical applicability. Regulatory status was considered product-, indication-, and jurisdiction-specific. Commercial availability alone was not considered as evidence of regulatory authorization or durable regenerative efficacy.

## 3. Biomaterials and Scaffold Design for Tissue Regeneration

**Figure 2:**
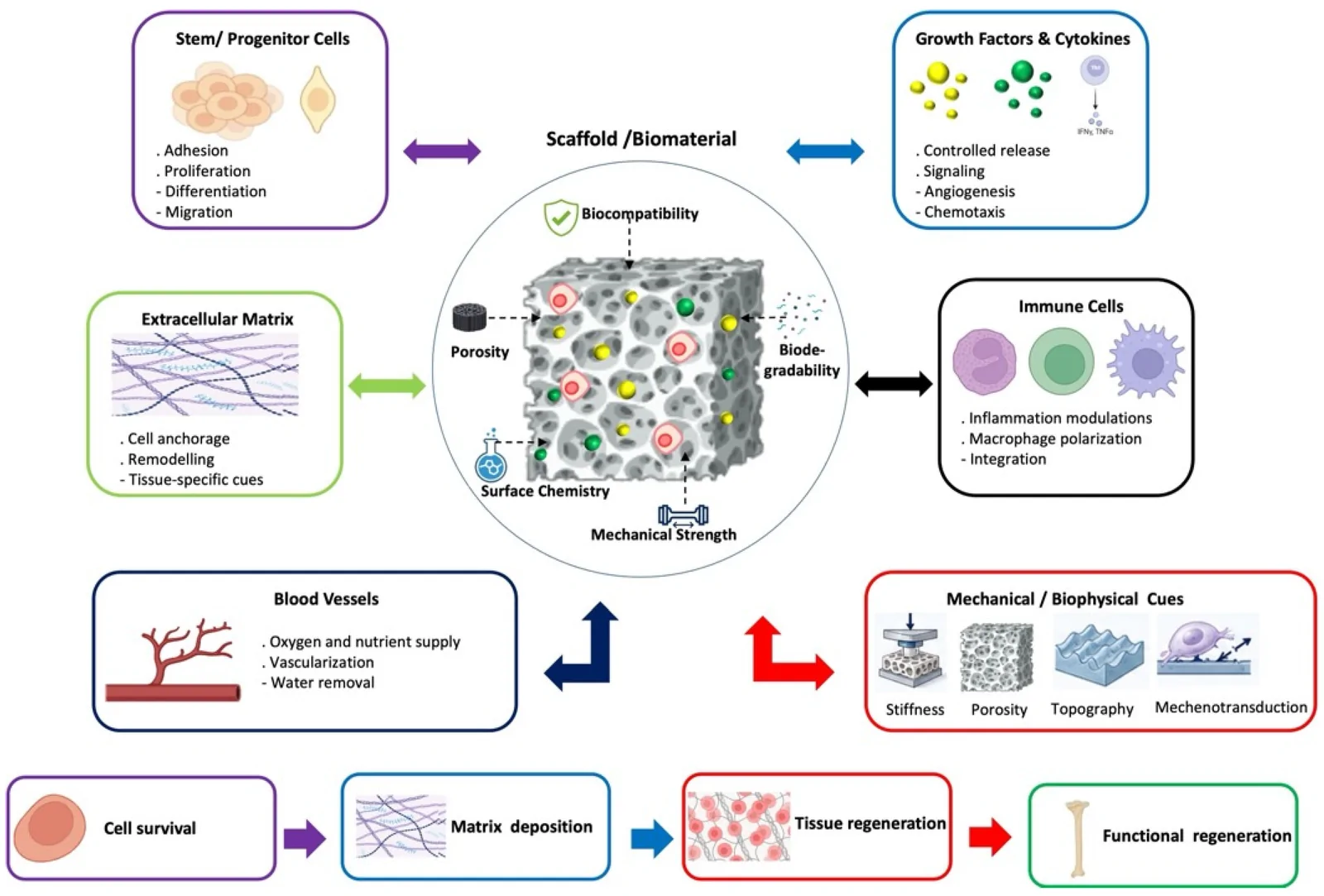
Scaffold–cell–bioactive signal interactions in tissue regeneration. Interactions between scaffolds, cells, extracellular matrix, soluble signals, immune cells, vascular components, and biophysical cues during tissue regeneration. Scaffold properties regulate cell survival, matrix deposition, tissue integration, and progression toward functional regeneration. The figure was created in Microsoft PowerPoint using graphical elements obtained from BioRender.com.

Biomaterials are central to tissue engineering because they provide physical support and biological cues for tissue repair (Figure 2). As illustrated in Figure 2, scaffold composition, porosity, surface chemistry, mechanical properties, and degradation behavior influence these interactions and ultimately determine cell survival, matrix deposition, tissue integration, and functional regeneration. Early scaffolds were mainly designed as temporary structural matrices, but modern biomaterials are increasingly bioactive, biodegradable, mechanically tunable, and capable of modulating cell and immune responses [12].

Natural biomaterials such as collagen, gelatin, fibrin, alginate, chitosan, hyaluronic acid, silk fibroin, and decellularized extracellular matrix are widely used because they resemble components of the native extracellular matrix [13] (Table 1). These materials often show good biocompatibility, cell adhesion, and biodegradability. Collagen and gelatin are particularly useful for skin, bone, cartilage, and vascular regeneration because they support cell attachment and matrix remodeling [14,15]. Hyaluronic acid-based materials are valuable for soft tissue regeneration because of their hydration capacity, viscoelasticity, and role in wound repair [16]. Decellularized extracellular matrix scaffolds are attractive because they preserve tissue-specific structural and biochemical signals [17].

Synthetic biomaterials such as polycaprolactone, polylactic acid, polyglycolic acid, poly(lactic-co-glycolic acid), polyethylene glycol, and polyurethane provide greater control over the mechanical properties, degradation rate, reproducibility, and fabrication [18]. However, synthetic materials often require modification with peptides, proteins, growth factors, or extracellular matrix components to improve cell adhesion and bioactivity.

Composite scaffolds combine natural and synthetic materials to balance biological performance and mechanical strength [19]. For example, hydrogel–polymer composites can provide a cell-friendly hydrated environment while maintaining sufficient structural stability [20]. Ceramic–polymer composites containing hydroxyapatite, β-tricalcium phosphate, or bioactive glass are widely investigated for bone regeneration because they mimic the mineral phase of bone [21].

Recent advances also include smart and stimuli-responsive biomaterials [22]. These materials respond to environmental signals such as pH, temperature, enzymes, reactive oxygen species, electrical stimulation, or mechanical stress [23]. Such responsiveness enables controlled drug release, dynamic changes in material properties, inflammation-responsive degradation, and localized delivery of regenerative signals. In plastic and reconstructive applications, these systems are particularly relevant to chronic and inflammatory wounds, scar-prone tissue environments, peripheral nerve repair, and soft-tissue reconstruction, where the biological requirements change dynamically during healing [24].

From a reconstructive perspective, biomaterial selection should therefore be driven by the clinical function required rather than by material class alone. Hydrated and compliant matrices are particularly suited to skin and soft-tissue defects, whereas mechanically reinforced composites are more appropriate for contour-bearing craniofacial and cartilage reconstruction [25]. In regenerative aesthetics, degradation kinetics, viscoelasticity, injectability, and the magnitude of the host inflammatory response are equally important because they influence tissue remodeling, fibrosis, softness, and long-term contour stability [26].

**Table 1:** Major biomaterials used in tissue engineering and regeneration.

Biomaterial	Type	Advantages	Degradation Profile	Mechanical Characteristics	Immunological/Manufacturing Considerations	Main Applications	References
Collagen	Natural	Biocompatible, ECM-like	Enzymatically degradable; degradation can be rapid unless crosslinked	Generally soft with limited intrinsic mechanical strength; often requires reinforcement	Low immunogenicity after appropriate purification; source variability, crosslinking, and sterilization may alter structure and bioactivity	Skin, bone, cartilage	[14,15]
Hyaluronic acid	Natural	Hydrating, bioactive	Rapid enzymatic degradation in native form; persistence can be increased by crosslinking	Soft and highly hydrated; limited load-bearing capacity unless chemically modified or reinforced	Generally biocompatible; molecular weight, crosslinking chemistry, residual reagents, and sterilization influence biological response	Skin, soft tissue	[16,27]
PLGA	Synthetic	Tunable degradation	Hydrolytically degradable; rate depends on lactide:glycolide ratio and molecular weight	Moderate and tunable mechanical properties; loses strength during degradation	Relatively reproducible manufacturing; acidic degradation products may alter the local microenvironment; surface modification is often required for cell adhesion	Drug delivery, bone	[28,29,30]
PCL	Synthetic	Strong, printable	Slowly hydrolytically degradable with prolonged persistence	Relatively high mechanical stability; suitable for structural reinforcement and 3D printing	Reproducible and readily fabricated; hydrophobic surface and slow degradation may limit cellular interaction and remodeling without modification	Bone, cartilage	[31,32]
Decellularized ECM	Natural/tissue-derived	Tissue-specific cues	Enzymatic degradation; strongly dependent on tissue source, processing, and crosslinking	Usually limited mechanical strength after decellularization; often requires reinforcement or blending	Residual DNA, cellular components, endotoxin, donor variability, decellularization, crosslinking, and sterilization require careful control	Organ and tissue repair	[17]

Note: ECM, extracellular matrix; PLGA, poly(lactic-co-glycolic acid); PCL, polycaprolactone; 3D, three-dimensional; DNA, deoxyribonucleic acid.

## 4. Biofabrication and 3D Bioprinting: Precision Is Not the Same as Function

Biofabrication has expanded the spatial vocabulary of tissue engineering, but precision in patterning is not equivalent to functional tissue formation [33]. For plastic and reconstructive applications, the clinically relevant question is whether geometric control can be translated into viable, vascularized, mechanically appropriate tissues that preserve barrier function, soft-tissue pliability, contour, or tissue-specific architecture after implantation. Extrusion printing, inkjet or droplet-based printing, laser-assisted bioprinting, stereolithography (SLA), digital light processing (DLP), and emerging volumetric approaches can organize cells and biomaterials with increasing geometric control, yet each platform operates within a distinct material-processing window [34]. Extrusion-based bioprinting is compatible with viscous, high-cell-density, and multicomponent hydrogel bioinks, but its success depends on shear-thinning behavior, gelation kinetics, extrusion pressure, and post-printing shape fidelity [35]. Increasing viscosity may improve structural stability, but excessive polymer concentration or extrusion stress can impair cell viability, migration, and differentiation. Inkjet-based approaches offer rapid and relatively high-resolution droplet deposition, but they are generally limited by low-viscosity formulations, lower cell densities, and potential thermal or mechanical stress during droplet generation [36]. Laser-assisted bioprinting can reduce nozzle-associated shear stress and enable precise cell placement, although technical complexity, cost, and material compatibility remain important limitations [37]. Light-based approaches such as SLA and DLP provide high-resolution fabrication through photocrosslinkable hydrogels, but their biological performance depends on photoinitiator selection, exposure dose, optical penetration, and cytocompatible crosslinking conditions [38].

This trade-off makes bioink design the functional bottleneck of 3D bioprinting. A successful bioink must simultaneously satisfy printability, mechanical stability, cytocompatibility, degradability, and biological instructiveness, rather than merely preserving the printed geometry [39]. Single-component hydrogels, including gelatin methacryloyl, alginate, collagen, fibrin, hyaluronic acid, chitosan, and other polysaccharide systems, provide useful matrices but often fail to balance shape fidelity with cell adhesion, migration, matrix deposition, and tissue-specific maturation [40]. Accordingly, recent work has shifted toward multicomponent and composite bioinks, including decellularized extracellular matrix (dECM)-containing formulations, ceramic- or bioactive glass-reinforced inks, nanocellulose-based systems, clay minerals, and other nanocomposites [41]. dECM-based bioinks are particularly attractive because they retain tissue-specific extracellular matrix components, growth factors, and biochemical cues that are difficult to reproduce using purified natural or synthetic polymers alone [42]. However, dECM bioinks often require further tuning of the viscosity, gelation rate, mechanical strength, and remodeling behavior before they can be reliably used for high-fidelity printing [43]. Nanocellulose and cellulose nanocrystals can function as rheological modifiers and reinforcing components, improving shear-thinning behavior, mechanical stability, and shape fidelity in hydrogel bioinks [44]. Clay minerals and nanosilicates are similarly used to modulate rheology, improve mechanical resistance, support printability, and provide additional bioactive or drug-loading functions [45]. Therefore, the key advance in biofabrication is not the ability to print more precise shapes, but the ability to print regenerative microenvironments in which geometry is integrated with biochemical, mechanical, and dynamic cues capable of supporting tissue remodeling.

The weakness of the field is that printed shape is still too often mistaken for biological maturity. In plastic and aesthetic surgery, a construct may reproduce the contour of skin, fat, or cartilage, but still fail to provide the biological functions that determine clinical outcome, including vascular ingrowth, dermal remodeling, pigmentation, appendage formation, soft-tissue pliability, long-term volume retention, and mechanical stability under facial or body movement [46,47]. This distinction is especially clear in bioprinted skin. Preclinical studies indicate that the layered deposition of keratinocytes, fibroblasts, and hydrogels can improve wound closure and dermal-epidermal organization, but these findings have been derived predominantly from experimental models, and current constructs remain far from routine clinical application [48,49]. Major unresolved limitations include stable vascularization, pigmentation, innervation, hair follicles, sweat glands, and restoration of the dynamic barrier function of native skin. For burns, chronic wounds, and postsurgical soft-tissue defects, in situ bioprinting is attractive because it can deposit autologous cells directly onto irregular wound beds, but the endpoint remains functional skin regeneration rather than simple surface coverage [50,51].

**Figure 3:**
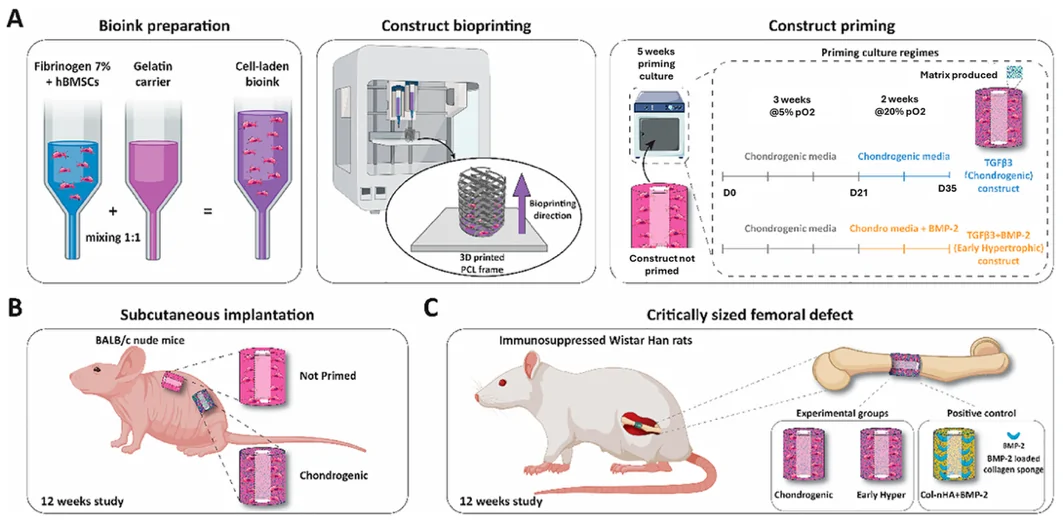
Schematic overview of bioprinted cartilaginous templates for orthopedic applications: (**A**) fabrication of a PCL-reinforced 3D-bioprinted cartilaginous template, followed by (**B**) subcutaneous implantation and (**C**) implantation into a critical-sized femoral defect. Reprinted from Refs. [57,58].

A similar limitation applies to adipose and soft-tissue reconstruction, where the goal is not only to fill a defect but to generate a living, vascularized, mechanically compliant tissue that maintains volume and integrates with the host [52,53]. Gelatin-, collagen-, hyaluronic acid-, alginate-, silk-, and dECM-based systems can provide cytocompatible matrices for adipose-derived cells, but their clinical value depends on vascular support, matrix remodeling, degradation kinetics, and resistance to contraction or resorption [54,55]. Cartilage bioprinting is also highly relevant to auricular, nasal, and craniofacial reconstruction, yet reproducing a patient-specific ear or nasal framework does not ensure chondrogenic maturation, elastic recoil, long-term shape retention, or integration with surrounding soft tissue [56]. As a proof-of-concept for spatially organized osteochondral regeneration, Pitacco et al. demonstrated a 3D-bioprinting strategy combining cartilaginous templates with BMP-2-mediated osteogenic cues, illustrating how spatially defined biological and mechanical signals can be integrated within a single construct (Figure 3) [57,58]. Although developed in an orthopedic setting, the same design principle is relevant to composite craniofacial reconstruction, where adjacent cartilage, bone, and soft-tissue interfaces require coordinated regeneration. Therefore, vascularization remains a decisive barrier in this field, not because every aesthetic construct must become an organ, but because skin flaps, fat graft-like constructs, and thick soft-tissue substitutes all require oxygen and nutrient transport to avoid necrosis, fibrosis, or volume loss.

Current strategies such as endothelial co-culture, sacrificial or coaxial channels, angiogenic factor delivery, prevascularized modules, and microfluidic conditioning can improve perfusion-related performance, but they do not yet make large, clinically reliable skin or soft-tissue constructs routine [59,60]. dECM-containing bioinks are particularly relevant for regenerative aesthetics because adipose-, skin-, and cartilage-derived matrices can retain tissue-specific biochemical cues that purified polymers cannot fully reproduce [17]. However, dECM bioinks often require reinforcement or blending because their weak mechanical properties and variable printability may limit structural fidelity in contour-bearing applications [35,61]. Thus, for plastic surgery and aesthetic medicine, the key benchmark should shift from whether a construct looks anatomically correct after printing to whether it can support durable wound repair, scar modulation, vascularized soft-tissue regeneration, and stable three-dimensional contour restoration after implantation.

These limitations suggest that the next phase of biofabrication in plastic and aesthetic surgery should be judged less by visual resemblance and more by functional performance. Standardized reporting should move beyond representative images to include post-printing cell viability, maturation markers, matrix deposition, mechanical properties, perfusion or angiogenic readouts, degradation behavior, immune response, and reproducibility across batches, printers, and operators. For skin, adipose, and cartilage-related applications, these endpoints should ultimately connect to clinically meaningful outcomes, including wound repair quality, scar modulation, soft-tissue pliability, vascularized volume retention, contour stability, and host integration. A printed construct that appears elegant in a figure but cannot be reproduced across laboratories or clinically relevant models remains a demonstration, not a regenerative platform.

## 5. Cell-Based Strategies in Regenerative Medicine

Cell-based therapy remains an important regenerative strategy, but its relevance to plastic and reconstructive surgery depends less on differentiation potential alone than on whether transplanted cells can improve vascularization, wound repair, scar remodeling, soft-tissue survival, and durable host integration. Cell-based therapy remains a major pillar of tissue engineering. Mesenchymal stem/stromal cells are among the most widely studied cells because they can be obtained from bone marrow, adipose tissue, umbilical cord, dental pulp, and other tissues [62]. In reconstructive settings, their principal value is increasingly attributed to trophic, angiogenic, and immunomodulatory signaling rather than the direct replacement of damaged tissue [63,64].

Adipose-derived stromal cells are particularly relevant to regenerative and aesthetic surgery because they can be obtained during liposuction and combined with autologous fat, dermal matrices, hydrogels, or platelet-derived products. In fat grafting, these cells are intended to support early graft survival by promoting angiogenesis, limiting inflammation, and assisting extracellular-matrix remodeling during the ischemic period after transplantation [65]. Cell-assisted approaches have also been investigated for facial rejuvenation, chronic ulcers, burn wounds, radiation fibrosis, and scar remodeling [66]. However, stromal vascular fraction, freshly isolated adipose-derived cells, culture-expanded adipose MSCs, microfat, and nanofat are biologically distinct preparations and should not be treated as equivalent interventions. Stromal vascular fraction is a heterogeneous mixture containing endothelial, immune, perivascular, and stromal populations, whereas expanded MSC products are selected more but are affected by culture-induced senescence and phenotypic drift [67]. Human clinical studies have reported improvements in fat-graft retention or facial skin quality with selected adipose-derived preparations [68]. However, the evidence remains heterogeneous and cannot be generalized across stromal vascular fraction, freshly isolated adipose-derived cells, culture-expanded MSCs, microfat, and nanofat. These interventions differ substantially in cell composition, processing, regulatory classification, and degree of clinical validation.

For cutaneous reconstruction, transplanted MSCs may contribute to re-epithelialization, granulation-tissue formation, neovascularization, and collagen remodeling [69]. Adipose-derived stromal cells have been incorporated into collagen-based dermal templates, platelet-rich plasma gels, gelatin microspheres, acellular dermal matrices, and bioprinted hydrogels [70]. Preclinical studies generally report faster wound closure, increased vascular density, improved collagen maturation, and reduced inflammatory or fibrotic responses [71]. However, these findings are derived predominantly from experimental models, and their reproducibility in clinically heterogeneous wounds remains to be established. Nevertheless, wound closure alone is an insufficient endpoint for plastic surgery. Studies should also assess epidermal architecture, adnexal regeneration, collagen alignment, pigmentation, sensory recovery, tissue elasticity, and hypertrophic scar formation [72]. This distinction is important because rapid matrix deposition does not necessarily produce functionally or aesthetically superior skin.

Induced pluripotent stem cells (iPSCs) provide a broader cellular repertoire than adult MSCs. Patient-derived iPSCs can be differentiated into keratinocytes, fibroblasts, melanocytes, endothelial cells, neural crest derivatives, adipogenic cells, osteoblasts, and chondrocytes, supporting disease modeling and the construction of patient-specific skin, cartilage, and craniofacial tissues [73]. Human pluripotent stem cell-derived skin organoids have generated stratified epidermis, dermis, hair follicles, sebaceous structures, and sensory-neuron-associated elements, demonstrating that complex skin appendages can be reproduced under controlled developmental conditions [74,75]. Layered constructs composed of iPSC-derived keratinocytes and fibroblasts have also formed skin-like tissues after transplantation in preclinical animal models. Together with in vitro organoid studies, these findings demonstrate biological feasibility but not clinical efficacy. iPSC-derived skin and craniofacial constructs therefore remain predominantly experimental, with routine reconstructive or aesthetic use limited by tumorigenicity, genomic stability, manufacturing complexity, and the absence of robust long-term clinical validation [76].

The translational risk profile of iPSCs is substantially different from that of adult stromal cells. Residual undifferentiated cells may form teratomas, whereas prolonged reprogramming and expansion may introduce genetic, epigenetic, or chromosomal abnormalities [77]. Additional concerns include incomplete lineage specification, immature tissue phenotype, variable differentiation efficiency, immunogenicity of derived products, lengthy manufacturing, and the cost of patient-specific quality control [78,79]. For reconstructive use, iPSC-derived cells must also retain stable identity after implantation and respond appropriately to mechanical loading, inflammation, ultraviolet exposure, and the wound microenvironment [80]. Accordingly, release criteria should include genomic integrity, residual pluripotency markers, differentiation purity, functional potency, and long-term tumorigenicity rather than viability alone [81].

Tissue-specific stem and progenitor cells offer a more restricted but often more controllable alternative. Epidermal stem cells and keratinocyte progenitors can restore epithelial coverage [82]. Hair follicle stem cells and dermal papilla populations are relevant to follicular regeneration [83]. Endothelial progenitor cells can support neovascularization [84], and chondroprogenitor and osteoprogenitor cells may be applied to auricular, nasal, mandibular, and craniofacial reconstruction [85]. Schwann cells and neural progenitors are also being investigated for peripheral nerve defects encountered after trauma, tumor resection, or reconstructive surgery [86]. Their narrower lineage potential may reduce inappropriate differentiation, but isolation yield, donor-site morbidity, loss of phenotype during expansion, and limited proliferative capacity remain important constraints [87].

Cell-sheet engineering addresses some of the cell-retention problems associated with suspension injection [88]. Cells are harvested as contiguous layers while preserving cell–cell junctions and cell-deposited extracellular matrix, allowing the sheet to attach directly to the recipient surface without a permanent synthetic scaffold [89]. In preclinical full-thickness wound models, adipose-derived stromal cell sheets have improved re-epithelialization, vascularization, and wound closure, with some studies also reporting reduced scar formation [90]. These findings should nevertheless be interpreted as preclinical evidence because clinically scalable implantation, fixation, vascularization, and long-term scar outcomes remain insufficiently established. Multilayer cell sheets may provide greater cell density and paracrine activity, but increasing thickness also aggravates oxygen and nutrient limitations before host vascular integration [91]. Mechanical fragility, contraction, difficult fixation to irregular wounds, and the absence of appendages or mature vascular networks further limit their use in large reconstructive defects [92].

Three-dimensional bioprinting extends cell-based therapy by controlling the spatial organization of keratinocytes, fibroblasts, endothelial cells, pericytes, melanocytes, and stromal cells within tissue-specific bioinks [93]. In preclinical animal models, vascularized multilayered skin constructs have achieved host–graft vascular connection, while more recent experimental systems have incorporated skin-derived extracellular matrix, microvascular fragments, and wound-matched geometries to improve integration [94,95]. Although these studies demonstrate feasibility, they should not be interpreted as evidence that living bioprinted skin has reached routine clinical readiness. For plastic surgery, this approach could support the customized coverage of irregular burns, traumatic defects, donor-site wounds, or oncologic resections [96]. Current constructs, however, rarely reproduce the full functional complexity of native skin, including stable perfusion, innervation, pigmentation, lymphatic drainage, sweat glands, sebaceous glands, and correctly oriented hair follicles [97,98]. Printing-associated shear stress, cytotoxic crosslinking, bioink variability, weak mechanical properties, diffusion limitations, sterilization, scalability, and batch-specific release testing remain major translational barriers [99].

The maturity of cell-based approaches therefore spans a wide translational spectrum. Autologous adipose tissue-based and selected stromal vascular fraction-assisted procedures have already entered clinical practice in some settings, although the protocols and evidence remain heterogeneous. Culture-expanded MSC products are supported by extensive preclinical research and selected early clinical investigations but remain indication- and jurisdiction-dependent. In contrast, iPSC-derived constructs, engineered cell sheets, and living bioprinted tissues remain predominantly experimental. These categories should not be discussed as clinically equivalent merely because they share a cellular component. Each requires defined cellular identity, dose, viability, potency, differentiation stability, biodistribution, immunogenicity, and long-term safety. In regenerative aesthetics, efficacy should also be judged by scar quality, tissue softness, surface texture, pigmentation, contour durability, and patient-reported appearance, not only by cell survival, wound closure, or histological collagen content.

## 6. Extracellular Vesicles and Cell-Free Regenerative Therapies

Stem and stromal cell therapies remain important in plastic surgery and regenerative aesthetics, but their therapeutic interpretation has shifted from cell replacement to paracrine regulation [100]. In cutaneous repair, scar modulation, soft-tissue remodeling, and hair restoration, many benefits once attributed to engraftment or differentiation are now understood as effects of secreted growth factors, cytokines, lipids, nucleic acids, metabolites, and extracellular vesicles [101,102,103]. This shift is clinically relevant because live-cell transplantation is constrained by limited survival after implantation, donor and passage variability, senescence, immune compatibility, storage, embolic risk, and regulatory uncertainty [104,105]. Cell-free products, including conditioned medium, total secretome, and extracellular vesicle-enriched preparations, therefore attempt to preserve the bioactive signaling of stem/stromal cells while reducing the complexity of administering viable cells [64,106].

For skin and soft-tissue regeneration, extracellular vesicles are particularly attractive because they can coordinate several repair processes at once [107,108]. In vitro and preclinical animal studies suggest that vesicle-based systems have been reported to promote keratinocyte migration, dermal fibroblast proliferation, endothelial tube formation, angiogenesis, macrophage polarization, collagen deposition, and extracellular matrix remodeling while reducing excessive inflammation and oxidative stress [109,110]. These effects are directly relevant to chronic wounds, diabetic ulcers, burns, surgical incisions, radiation-related fibrosis, post-procedure recovery, and aesthetic skin rejuvenation [110,111]. However, the same activities require careful interpretation. Increased fibroblast activity or collagen production may support dermal regeneration, but it may also be undesirable in a profibrotic microenvironment unless collagen organization, α-SMA expression, TGF-β/Smad signaling, and scar quality are evaluated [112].

Adipose-derived vesicles are especially relevant to regenerative aesthetics because adipose tissue is already central to plastic surgery through liposuction, fat grafting, and soft-tissue contouring [53,113]. Predominantly in vitro and preclinical animal studies of ASC- or ADSC-derived exosomes have been shown to enhance fibroblast proliferation and migration, stimulate collagen-related gene expression, improve re-epithelialization, increase vascularization, and accelerate wound closure [114,115]. When combined with hyaluronic acid, collagen, platelet-rich plasma, or decellularized extracellular matrix hydrogels, vesicles are no longer used as a simple topical biologic; they become part of a bioactive delivery system [116,117]. This is important for aesthetic and reconstructive applications because hydrogels and scaffolds can improve local retention, protect vesicle cargo, prolong release, and provide a matrix-like environment for tissue remodeling [118,119].

The same logic extends to hair restoration and skin biomodulation [74]. Our previous study showed that in ex vivo human hair follicle culture, umbilical cord blood-derived extracellular vesicles have been reported to promote hair shaft elongation, preserve hair bulb diameter, delay anagen-to-catagen transition, and increase laminin V and collagen XVII expression, suggesting potential effects on the follicular basement membrane and stem cell niche [120]. In photoaging or inflammatory skin damage, vesicle-based approaches may act through antioxidant signaling, immune modulation, angiogenesis, and matrix preservation [121,122]. Nevertheless, ex vivo or small-animal findings do not establish clinical efficacy. Human clinical evidence for extracellular-vesicle-based wound, hair, and aesthetic interventions remains substantially less mature than the preclinical literature, and commercial availability should not be interpreted as evidence of regulatory authorization or therapeutic efficacy. Regulatory status also varies among jurisdictions and intended uses. For scalp and aesthetic skin applications, unresolved issues include tissue penetration, follicular targeting, repeat dosing, long-term safety, and durability of response.

The main barrier to translation is product definition rather than biological plausibility. Vesicle preparations differ according to donor source, cell type, passage number, culture medium, oxygen tension, preconditioning, isolation method, storage, purity, cargo composition, and dose metric [123]. Ultracentrifugation, precipitation, size-exclusion chromatography, filtration, and microfluidic isolation do not generate interchangeable products [124]. Therefore, a claim that “exosomes promote regeneration” is too imprecise unless the vesicle source, identity markers, particle size and number, protein contamination, potency assay, biodistribution, and release profile are clearly defined [125,126]. In future studies, potency assays should match the intended plastic surgery indication. Wound products should test re-epithelialization, angiogenesis, inflammation, and bacterial control. Scar-modulating products should assess α-SMA, collagen I/III balance, matrix organization, and tissue stiffness. Hair products should evaluate follicular delivery, anagen maintenance, dermal papilla activity, and follicle stem cell markers.

Thus, the most credible future for cell-free therapy in regenerative aesthetics is not as a vague substitute for stem cells, but as a defined biological product integrated with an appropriate delivery platform. Scaffold- and hydrogel-mediated systems may improve retention and controlled release, but they also create combination products that are harder to standardize, regulate, and compare across studies [127,128]. Clinical translation will require reproducible manufacturing, stable formulations, indication-specific potency assays, and outcome measures that reflect not only wound closure or collagen staining, but also scar quality, skin texture, soft-tissue durability, and functional cosmetic restoration.

## 7. Immunomodulation in Tissue Engineering

The host immune response is particularly important in plastic and reconstructive surgery because it determines whether an implanted or injected material undergoes constructive integration or progresses toward chronic inflammation, fibrosis, contracture, granuloma formation, or clinically unacceptable scar remodeling [129]. In skin and soft-tissue repair, neutrophils and monocytes provide early antimicrobial defense and debris clearance, while macrophages subsequently regulate angiogenesis, fibroblast recruitment, extracellular matrix deposition, and wound contraction [130]. This response must be transient and appropriately resolved. Sustained production of TNF-α, IL-1β, IL-6, reactive oxygen species, and proteolytic enzymes can impair re-epithelialization, destabilize newly formed vessels, and maintain fibroblasts in a profibrotic state, contributing to chronic wounds, hypertrophic scars, delayed graft incorporation, and peri-implant fibrosis [131].

Macrophages are central to this transition. Early inflammatory populations support phagocytosis and host defense, whereas later reparative populations promote efferocytosis, vascularization, matrix remodeling, and inflammation resolution [132]. The M1/M2 framework remains useful as a descriptive model, but macrophage states in vivo are heterogeneous and temporally dynamic [133]. Moreover, prolonged “M2-like” signaling is not uniformly beneficial, because selected reparative subsets may enhance TGF-β activity, myofibroblast differentiation, excessive collagen deposition, and foreign-body capsule formation [134]. Immunomodulatory strategies should therefore regulate the timing and magnitude of macrophage responses rather than simply increase M2-marker expression.

This principle is particularly relevant to injectable fillers, acellular dermal matrices, tissue-engineered skin, meshes, breast implants, and fat-grafting adjuncts. After implantation, protein adsorption establishes the initial biological interface, while material chemistry, wettability, stiffness, roughness, pore structure, particle morphology, and degradation products influence macrophage adhesion, phagocytosis, foreign-body giant-cell formation, and vascular infiltration [135,136,137]. In biodegradable collagen-stimulating fillers, a controlled foreign-body response contributes to fibroblast activation and neocollagenesis [138]. However, excessive or uneven inflammation may produce nodules, granulomas, localized stiffness, or irregular fibrosis. Particle size distribution, aggregation, surface architecture, injection depth, local concentration, and degradation kinetics should therefore be assessed together with macrophage behavior and collagen organization [139].

Hydrogels and scaffolds can provide localized immune regulation by delivering IL-4, IL-10, antioxidants, pro-resolving lipid mediators, macrophage-modulating peptides, nucleic acids, or extracellular vesicles [140,141]. Release kinetics are critical because premature suppression may compromise debridement and antimicrobial defense, whereas prolonged exposure may impair normal immune competence or promote abnormal remodeling [142]. Stimuli-responsive systems triggered by reactive oxygen species, proteases, pH, or temperature may provide more appropriate temporal control [143,144]. MSC-derived extracellular vesicles are also being incorporated into hydrogels to improve local retention and simultaneously modulate inflammation, angiogenesis, fibroblast activity, and matrix deposition, which is relevant to chronic wounds, burns, donor-site healing, and post-procedural skin repair [116,145,146].

Decellularized matrices should likewise be regarded as immune-active rather than biologically inert [147,148]. Their performance depends on tissue source, residual DNA and endotoxin, crosslinking, sterilization, and degradation rate [149]. Incomplete decellularization or excessive crosslinking can prolong macrophage activation and favor fibrous encapsulation instead of integration. Evaluation should therefore extend beyond CD86/CD206 staining to include temporal immune-cell profiles, cytokine expression, vascularization, myofibroblast activation, collagen alignment, capsule thickness, tissue stiffness, and scar quality [150]. For reconstructive and aesthetic surgery, clinically meaningful outcomes also include implant softness, contour stability, skin texture, and the absence of palpable nodules or contracture.

## 8. Organoids and Organ-on-a-Chip Systems

Organoids are three-dimensional, self-organizing systems derived from stem or progenitor cells that reproduce selected structural and functional features of native tissues [151,152]. Within plastic, reconstructive, and aesthetic medicine, their principal relevance lies in skin and skin-appendage models that can reproduce epithelial–mesenchymal interactions and selected features of pigmentation, hair follicles, sensory structures, and vascular niches [153].

Skin organoids provide a more tissue-relevant experimental system than monolayer cultures because they preserve epithelial–mesenchymal interactions and permit the partial reconstruction of skin architecture [154]. Human-induced pluripotent stem cell (hiPSC)-derived organoids can develop a stratified epidermis and dermal compartment together with pigmented hair follicles, sebaceous glands, Merkel cells, neural elements, adipocytes, and in optimized systems, eccrine sweat glands [155,156]. These structures arise through the coordinated regulation of TGF-β, FGF, and BMP signaling, although differentiation efficiency and off-target lineage formation remain dependent on the hiPSC line and culture protocol.

For plastic and reconstructive surgery, the relevance of these models extends beyond wound coverage [157]. Cultured epithelial autografts can provide epidermal closure in extensive burns but do not restore a functional dermis or skin appendages [158]. Organoid-derived constructs may therefore offer a route toward skin substitutes that better reproduce pigmentation, sensation, thermoregulation, sebaceous function, and hair growth. Hair-bearing organoids are also being investigated as sources of follicular units for alopecia, while patient-derived skin organoids could support the individualized assessment of abnormal scarring, pigmentary disorders, inherited skin diseases, and responses to regenerative treatments [159]. Nevertheless, current organoids should be regarded primarily as experimental models rather than transplant-ready skin because their architecture still resembles developing tissue more closely than mature adult skin.

Skin organoids are also relevant to regenerative aesthetics [160]. Aging models can be generated through prolonged culture, senescence induction, genetic manipulation, or repeated exposure to ultraviolet radiation and environmental pollutants [161]. Relevant readouts include epidermal thickness, barrier integrity, keratinocyte proliferation, cellular senescence, reactive oxygen species, matrix metalloproteinase activity, and collagen or elastin organization. Such systems can be used to evaluate antioxidants, retinoids, senolytics, anti-glycation agents, peptides, hyaluronic acid-based formulations, and other skin-modulating products. They may also help examine the cellular effects of laser resurfacing, microneedling, and chemical peeling, although standardized exposure protocols and clinically meaningful dose equivalence remain insufficiently established [162].

Vascularization remains a major constraint because diffusion alone cannot support the metabolic demands of larger constructs or ensure rapid graft integration [163]. Simply adding endothelial cells may not be sufficient. In one recent study, endothelial colony-forming cells produced capillary-like structures but interfered with epidermal stratification and hair follicle morphogenesis [164]. In contrast, combining hiPSC-derived skin organoids with vascular organoids in an air–liquid interface system generated more complex vascularized skin organoids containing endothelial cells, pericytes, vascular smooth muscle-like cells, and perifollicular vessels while preserving epidermal differentiation and hair follicle formation. The same system also contained CD45-positive hematopoietic cells, macrophages, CD207-positive immune cells, and neutrophils [164]. This illustrates that vascularization should reproduce a multicellular vascular niche rather than introduce endothelial cells alone [165].

Organ-on-a-chip platforms provide complementary control over perfusion, biochemical gradients, tissue interfaces, and environmental exposure. In skin-on-chip systems, endothelialized microchannels can deliver nutrients and permit the analysis of vascular permeability, inflammatory-cell recruitment, barrier transport, and tissue responses under flow [166]. Microfluidic exposure is particularly useful for modeling repeated contact with pollutants or topical agents and for evaluating wound dressings, transdermal delivery systems, microneedles, and other skin-directed technologies [167]. Wound- and fibrosis-oriented chips may further allow for the quantitative measurement of re-epithelialization, fibroblast contraction, endothelial dysfunction, inflammatory signaling, and TGF-β-dependent matrix remodeling [168]. However, current microfluidic systems do not yet reproduce a complete capillary network, lymphatic drainage, systemic metabolism, or the full mechanical environment of human skin.

Clinical translation remains limited by prolonged maturation, variability among cell lines and batches, dependence on poorly defined matrices such as Matrigel, and the absence of standardized manufacturing and quality-control criteria. Additional concerns include incomplete regional specification, limited immune and neural maturation, uncertain host integration, and the tumorigenic and genomic risks associated with residual pluripotent cells. For reconstructive applications, model performance should ultimately be judged by barrier restoration, vascular perfusion, appendage formation, mechanical integrity, and host integration. For regenerative-aesthetic applications, pigmentation, matrix organization, surface quality, and durability are also relevant. At present, skin organoids and organ-on-a-chip systems should therefore be regarded primarily as experimental modeling and testing platforms rather than clinically deployable regenerative products.

## 9. Nanotechnology in Tissue Engineering

Nanotechnology enables the design of biomaterials that approximate the nanoscale organization of the extracellular matrix [169]. Electrospun nanofibers are particularly relevant to skin and soft-tissue engineering because their high surface area and collagen-like architecture support cell adhesion, migration, and directional organization [170]. In wound dressings, dermal substitutes, scar-modulating matrices, and nerve guidance conduits, the fiber diameter, alignment, porosity, and degradation rate can be adjusted to influence cellular infiltration and matrix deposition [171].

Nanoparticles provide the localized delivery of drugs, growth factors, proteins, and nucleic acids [172]. Polymeric and lipid nanoparticles can protect labile cargo and prolong tissue retention, while hydroxyapatite or bioactive glass nanoparticles may improve osteoconductivity in craniofacial reconstruction [173,174]. Antimicrobial nanoparticles, including silver-based systems, have been incorporated into wound dressings to reduce bacterial burden [175]. However, excessive ion release may impair keratinocytes, fibroblasts, and vascular cells [176].

Conductive nanomaterials, such as graphene derivatives, gold nanostructures, carbon nanotubes, and conductive polymers, have been investigated predominantly in in vitro and preclinical models of peripheral nerve and muscle regeneration [177,178]. Although their electrical and topographical properties may promote neurite extension and cell alignment experimentally, clinical translation remains limited by uncertain biodegradation, tissue persistence, dose-dependent cytotoxicity, and limited long-term human evidence [179].

For plastic and regenerative aesthetic applications, nanomaterial performance should therefore be assessed together with particle size, surface charge, release kinetics, immune response, biodistribution, and long-term accumulation. Their value lies in improving local biological control without introducing persistent inflammation or material-related toxicity.

## 10. Artificial Intelligence and Digital Technologies

Artificial intelligence (AI) and machine learning should be regarded primarily as emerging enabling technologies rather than regenerative interventions themselves. They are increasingly used to analyze biomaterial, imaging, cellular, and manufacturing data and may support scaffold design, wound-healing prediction, scar assessment, skin-aging analysis, and the selection of patient-specific regenerative strategies in plastic and regenerative-aesthetic surgery [180,181].

For biomaterial development, machine-learning models can relate composition and processing variables to stiffness, porosity, swelling, degradation, cell compatibility, and printability [182]. In cell-based systems, automated image analysis can quantify cell morphology, viability, confluence, differentiation, and batch-to-batch consistency [5]. Digital analysis of clinical photographs, three-dimensional surface scans, ultrasound, or histological images may also improve the objective evaluation of wound closure, scar thickness, pigmentation, skin texture, and soft-tissue volume [183].

In 3D bioprinting, artificial intelligence can optimize bioink formulation, nozzle pressure, printing speed, temperature, and crosslinking conditions [184]. Real-time imaging and sensor feedback may identify filament collapse, nozzle obstruction, or geometric deviation during fabrication [185]. Medical imaging can further be used to generate patient-specific models for craniofacial defects, breast reconstruction, auricular reconstruction, and customized skin or soft-tissue scaffolds [186]. However, technical optimization or high predictive accuracy alone does not establish clinical utility. AI-assisted systems must demonstrate that their outputs improve reproducibility, decision-making, safety, or patient-relevant outcomes.

The major limitations are dataset quality, inconsistent experimental protocols, limited external validation, and poor model interpretability. Models developed from small or highly selected datasets may perform well internally but fail when applied to different institutions, devices, patient populations, or clinical workflows. External, and where feasible, prospective validation should therefore assess not only the discrimination or classification accuracy, but also calibration, robustness, reproducibility, and clinically relevant failure modes. Dataset diversity is particularly important in reconstructive and aesthetic applications because performance may vary with age, sex, skin phototype and ethnicity, wound etiology and severity, anatomical site, imaging device, lighting conditions, acquisition protocol, and clinical setting [187]. These variables should be represented in training and validation datasets and examined through prespecified subgroup analyses to reduce systematic bias.

Clinical deployment also requires attention to interpretability, governance, and regulatory oversight. The degree of explainability required should reflect the intended use: an algorithm used for exploratory biomaterial screening does not carry the same clinical consequence as one used to assess wounds, recommend treatment, determine manufacturing release, or guide patient-specific reconstruction [188]. Data provenance, model version control, human oversight, and post-deployment performance monitoring are therefore important when AI outputs may influence clinical decisions or product quality. Clinically deployable endpoints should ultimately be linked to measurable improvements in wound assessment, scar evaluation, patient-specific reconstruction, manufacturing consistency, treatment selection, or procedural planning rather than algorithmic accuracy alone [189]. AI-assisted systems should therefore complement, rather than replace, biological validation and clinical judgment.

AI-assisted systems should therefore complement, rather than replace, biological testing and clinical judgment. In reconstructive and aesthetic applications, their value should ultimately be demonstrated through clinically meaningful improvements in wound assessment, scar evaluation, patient-specific reconstruction, objective skin-quality analysis, manufacturing reproducibility, or procedural planning rather than predictive accuracy alone.

## 11. Tissue-Specific Applications

Tissue-engineering strategies must be adapted to the structural, biological, and clinical requirements of each target tissue. The following subsections focus on tissues with direct relevance to plastic, reconstructive, craniofacial, and aesthetic practice, including skin, bone, cartilage, peripheral nerve, vascularized soft tissue, and oral–craniofacial structures. Their principal regenerative strategies, clinically relevant goals, and remaining translational limitations are summarized in Table 2.

### 11.1. Skin and Wound Healing

Skin remains one of the most active targets of tissue engineering because burns, chronic wounds, diabetic ulcers, surgical defects, and traumatic injuries require the restoration of both barrier function and dermal architecture [190,191]. Current strategies include collagen and hydrogel scaffolds, decellularized dermal matrices, antimicrobial dressings, the controlled delivery of growth factors, stem/stromal cells, extracellular vesicles, and bioprinted skin constructs [192,193]. Effective substitutes should support keratinocyte migration, fibroblast-mediated matrix formation, vascular ingrowth, moisture balance, and infection control while limiting wound contraction and pathological scarring [194]. However, most engineered skin products still incompletely reproduce pigmentation, innervation, lymphatic drainage, and appendages such as hair follicles and sweat glands.

### 11.2. Bone Regeneration

Bone tissue engineering combines osteoconductive matrices, osteoinductive signals, and osteogenic cells [195]. Hydroxyapatite, β-tricalcium phosphate, bioactive glass, collagen, polycaprolactone, and their composites are frequently used to provide structural support and mineralized surfaces [196,197]. In plastic and craniofacial surgery, three-dimensional printing is particularly valuable for the patient-specific reconstruction of calvarial, mandibular, maxillary, and orbital defects [198]. Major challenges include the vascularization of large constructs, mechanical integration with host bone, infection control, and the avoidance of excessive or ectopic bone formation when potent osteogenic factors such as BMPs are used [199].

### 11.3. Cartilage Regeneration

Cartilage regeneration is particularly relevant to auricular, nasal, and other craniofacial reconstructive defects, where long-term shape stability, elasticity, and integration with surrounding soft tissue are essential. Regeneration remains difficult because mature cartilage is avascular and has limited intrinsic repair capacity [200]. Investigated approaches include autologous chondrocytes, mesenchymal stromal cells, cartilage-derived extracellular matrix, hydrogels, and spatially organized scaffolds [201,202]. The principal objective is to generate stable hyaline or elastic cartilage rather than fibrocartilage. Appropriate mechanical stimulation, nutrient diffusion, zonal organization, and the control of chondrocyte hypertrophy are therefore essential for long-term shape retention and functional maturation.

### 11.4. Nerve Regeneration

Peripheral nerve injuries are common after trauma, tumor resection, and reconstructive procedures. Nerve guidance conduits, aligned nanofibers, Schwann cells, neurotrophic factors, conductive biomaterials, and extracellular vesicles have been investigated to direct axonal extension and improve target reinnervation [203,204]. For clinically relevant defects, regeneration also requires vascularization, the suppression of excessive fibrosis, and the coordinated recovery of sensory and motor function [205].

### 11.5. Vascular Regeneration

Vascularization is a central requirement in reconstructive tissue engineering because implanted skin, adipose tissue, bone, cartilage-containing composites, and other thick constructs require rapid access to oxygen and nutrients [206]. Although engineered small-diameter vascular grafts remain an important field, the more immediate challenge for plastic and reconstructive applications is the formation of perfusable microvascular networks that can rapidly connect with host vessels. Current strategies therefore include endothelial-cell incorporation, angiogenic-factor delivery, prevascularized modules, microchannel formation, and biomaterials designed to promote host vascular ingrowth [207]. Clinically, the relevant endpoints are not vessel density alone but rapid perfusion, graft survival, reduced necrosis and fibrosis, and durable integration of the reconstructed tissue.

### 11.6. Dental and Periodontal Regeneration

Dental and periodontal engineering targets pulp–dentin regeneration, periodontal ligament restoration, cementum formation, and alveolar bone repair [208]. Dental pulp stem cells, periodontal ligament stem cells, stem cells from the apical papilla, and cells from exfoliated deciduous teeth are commonly combined with collagen, calcium phosphate, hydroxyapatite, or injectable hydrogels [209]. For craniofacial and oral reconstruction, successful regeneration requires the coordinated restoration of mineralized tissue, soft-tissue attachment, vascularization, and where relevant, sensory innervation [210].

### 11.7. Biomodulators and Skinboosters in Skin Regeneration

Biostimulatory injectables and skin-quality treatments occupy a distinct translational position within regenerative aesthetics because several products are already used clinically, while the evidence supporting a broader regenerative mechanism varies substantially among material classes and formulations [211]. Calcium hydroxyapatite, poly-L-lactic acid, polycaprolactone, and related biodegradable materials induce a controlled host response that can increase collagen deposition and dermal thickness, but the magnitude, organization, and durability of remodeling are product- and protocol-dependent [212]. In contrast, skin-quality injectables include heterogeneous formulations based on non-crosslinked or lightly crosslinked hyaluronic acid, polynucleotides, polydeoxyribonucleotide, amino acids, peptides, or other bioactive components [213]. Their commercial or clinical use should therefore be distinguished from evidence demonstrating sustained extracellular-matrix regeneration.

Their biological effects depend on interactions with fibroblasts, immune cells, endothelial cells, and the extracellular matrix. Hyaluronic acid mainly improves hydration and viscoelasticity while also providing a provisional matrix for cell migration [214]. However, these short-term physicochemical effects should be distinguished from evidence of neocollagenesis or durable extracellular-matrix remodeling. Polynucleotide- and polydeoxyribonucleotide-based formulations have been investigated in in vitro, ex vivo, preclinical, and human clinical studies for effects on fibroblast activity, angiogenic signaling, oxidative stress, and inflammation [215,216]. Particulate biostimulators act through material-dependent tissue responses in which particle morphology, size distribution, degradation rate, and local concentration influence macrophage recruitment, fibroblast activity, and subsequent matrix remodeling [217].

Regulatory and commercial status should also be interpreted at the level of the specific product and approved indication [218]. Selected hyaluronic acid, CaHA, and PLLA injectables have regulator-authorized aesthetic indications in certain jurisdictions, whereas other biostimulatory or biomodulatory formulations may be commercially available under different regulatory classifications. Importantly, authorization for the correction of wrinkles, contour, skin smoothness, or other aesthetic endpoints should not automatically be interpreted as regulatory validation of a broader claim of tissue regeneration or durable neocollagenesis.

These products are used for photoaged or thinned skin, fine wrinkles, acne scars, skin laxity, and anatomically delicate regions such as the neck, hands, and periorbital area [219]. These products are also combined clinically or experimentally with microneedling, lasers, radiofrequency, ultrasound-based devices, platelet-derived preparations, or extracellular-vesicle-based formulations [220,221]. The level of evidence for these combinations is highly variable. Biological plausibility or commercial availability should not be interpreted as evidence that additive or synergistic clinical benefit has been established, particularly for extracellular-vesicle-based aesthetic products.

Clinical outcomes depend on product composition, injection depth, dose, treatment interval, anatomical site, and patient factors [222]. Potential complications include edema, bruising, infection, nodules, granulomatous reactions, vascular compromise, irregular fibrosis, and overcorrection [223]. Future studies should clearly report whether evidence is derived from mechanistic experiments, controlled clinical trials, observational clinical use, or post-marketing experience. Standardized measures of dermal thickness, elasticity, hydration, collagen organization, pigmentation, texture, durability, and patient-reported improvement are also required to distinguish temporary skin-quality improvement from durable biological remodeling. This would better connect aesthetic skin treatment with the broader principles of controlled tissue regeneration.

**Table 2:** Tissue-specific applications of tissue engineering and regeneration.

Tissue/Application	Main Strategy	Representative Biomaterials/Cells	Regenerative Goal	Key Clinical Outcome Measures	Major Challenge	References
Skin and wound healing	Hydrogels, EVs, cell sheets, bioactive dressings	Collagen, HA, fibrin, MSCs, EVs	Re-epithelialization, angiogenesis, scar reduction	Wound closure, epithelial integrity, vascularization, infection control, scar quality, elasticity, pigmentation	Infection control and scar formation	[192,193,194]
Bone regeneration	3D scaffolds, osteogenic cells, BMP delivery	HA, TCP, bioactive glass, PCL, MSCs	New bone formation and defect repair	Bone volume and union, vascularization, mechanical integration, maintenance of craniofacial contour	Vascularization and mechanical strength	[195,196,197,198,199]
Cartilage regeneration	Injectable hydrogels, chondrocytes, zonal scaffold design	GelMA, collagen, cartilage ECM, MSCs	Hyaline-like cartilage restoration	Cartilage composition, mechanical properties, integration, shape retention, surface/contour stability	Fibrocartilage formation and poor integration	[200,201,202]
Nerve regeneration	Guidance conduits, aligned fibers, neurotrophic delivery	Schwann cells, neural stem cells, conductive polymers, EVs	Axonal growth and functional recovery	Axonal continuity, electrophysiology, sensory and motor recovery, muscle reinnervation	Inflammation, glial scarring, and limited reconnection	[203,204,205]
Vascular regeneration	Endothelialization, antithrombotic coatings, microvascular networks	Endothelial cells, biodegradable polymers, vascular grafts	Blood vessel replacement and perfusion support	Perfusion, vessel patency, host–graft vascular integration, tissue survival	Thrombosis and long-term patency	[206,207]
Dental and periodontal regeneration	Stem cell therapy, mineralized scaffolds, injectable matrices	DPSCs, PDLSCs, collagen, calcium phosphate, HA	Pulp, dentin, ligament, cementum, and alveolar bone repair	Mineralized tissue formation, periodontal attachment, vascularization, sensory recovery	Complex tissue interface regeneration	[208,209,210].
Aesthetic skin regeneration	Biomodulators, skinboosters, combination procedures	HA, CaHA, PLLA, PCL, PN/PDRN, peptides	Dermal hydration, collagen stimulation, ECM remodeling	Hydration, dermal thickness, elasticity, collagen organization, pigmentation, texture, durability, patient-reported improvement	Protocol standardization, objective endpoints, and safety	[211,212,213]
Soft-tissue reconstruction	Fat grafting adjuncts, hydrogels, dECM scaffolds, cell-assisted and vascularization strategies	Adipose tissue, ADSCs/SVF, dECM, collagen, HA	Vascularized and durable soft-tissue restoration	Volume retention, tissue pliability, contour stability, vascularization, fibrosis, host integration, symmetry	Resorption, insufficient vascularization, fibrosis, and unstable long-term contour	[224,225,226]

Note: 3D, three-dimensional; ADSCs, adipose-derived stem cells; BMP, bone morphogenetic protein; CaHA, calcium hydroxyapatite; dECM, decellularized extracellular matrix; DPSCs, dental pulp stem cells; ECM, extracellular matrix; EVs, extracellular vesicles; GelMA, gelatin methacryloyl; HA, hyaluronic acid; HAp, hydroxyapatite; MSCs, mesenchymal stem/stromal cells; PCL, polycaprolactone; PDRN, polydeoxyribonucleotide; PDLSCs, periodontal ligament stem cells; PLLA, poly-L-lactic acid; PN, polynucleotide; SVF, stromal vascular fraction; β-TCP, beta-tricalcium phosphate.

## 12. Clinical Translation and Commercialization

Despite major scientific progress, the clinical translation of regenerative technologies requires more than a demonstration of biological efficacy. A clinically viable product must combine reproducible biological performance with controlled manufacturing, defined product specifications, sterility, storage stability, transportability, regulatory compliance, cost-effectiveness, and practical integration into surgical or outpatient workflows [2,227]. These requirements differ substantially among acellular scaffolds, living-cell products, extracellular-vesicle formulations, biofabricated tissues, and combination products. Translation should therefore begin with a definition of the intended clinical use and the critical quality attributes (CQAs) that are most closely linked to product safety and performance.

CQAs and release criteria should be platform-specific and defined using prespecified acceptance ranges. For scaffolds, relevant attributes include composition, mechanical properties, degradation behavior, residual processing reagents, and microbiological quality; cell-based products additionally require the control of identity, purity, viability, dose, differentiation status, and where relevant, genomic stability. Extracellular-vesicle products require the characterization of source cells, particle concentration and size distribution, identity markers, purity, and storage stability, whereas biofabricated products additionally require the reproducibility of printing, cell distribution, and structural integrity. Potency assays should reflect the proposed mechanism of action and intended indication rather than relying on cell viability or particle number alone. Sterility, endotoxin or bioburden control, and batch comparability should likewise be integrated into manufacturing, with relevant process changes triggering the reassessment of product comparability.

Clinical evaluation should extend beyond short-term biological responses to indication-specific durability and long-term safety. Reconstructive applications should assess sustained defect correction, vascular and host integration, mechanical or sensory function where relevant, scar quality, and freedom from progressive fibrosis or resorption. Regenerative-aesthetic applications should additionally evaluate the persistence of volume or contour, tissue pliability, skin texture and elasticity, and patient-reported improvement. Histological collagen deposition alone should not be considered as evidence of durable regeneration. Post-market surveillance should therefore capture delayed infection, immune or fibrotic reactions, granuloma or nodule formation, vascular complications, ectopic tissue formation, persistent material accumulation, and adverse effects associated with repeated treatment, with product and lot traceability wherever feasible.

Regulatory classification remains particularly complex for combination products incorporating cells, biomaterials, biologics, genes, extracellular vesicles, or devices [228,229]. Product development should therefore consider the likely regulatory pathway early enough to influence material selection, manufacturing strategy, analytical testing, and clinical study design rather than treating regulation as a final step after efficacy has been demonstrated. Cost and accessibility must also be considered. Highly personalized regenerative therapies may require patient-specific cells, imaging, fabrication, specialized facilities, and prolonged quality-control procedures, whereas scalable off-the-shelf products require sufficient reproducibility across heterogeneous patients and treatment sites. Ultimately, successful translation will depend on balancing biological sophistication with manufacturability, quality assurance, clinical practicality, and demonstrable long-term patient benefit [230,231].

Importantly, translational maturity should not be inferred from commercial availability alone (Table 3). Regulatory authorization applies to a defined product, indication, manufacturing process, and jurisdiction, whereas commercial use may occur under substantially different regulatory pathways. Conversely, authorization of an injectable material for an aesthetic indication does not necessarily validate a proposed regenerative mechanism or long-term tissue-remodeling claim. This distinction is particularly important for extracellular-vesicle products, stromal-cell preparations, polynucleotide/PDRN formulations, skin-quality injectables, and biostimulatory materials. Regulatory status should therefore be reported separately from the level of clinical evidence and from evidence supporting the proposed mechanism of action.

## 13. Current Challenges and Future Perspective

Despite rapid methodological advances, the central challenge is to convert increasingly complex regenerative systems into reproducible and clinically meaningful therapies. Biomaterials, cells, extracellular vesicles, molecular cues, and fabrication processes are often optimized separately, although their interactions ultimately determine therapeutic performance. Changes in scaffold stiffness, degradation, cell source, cargo loading, or manufacturing conditions may alter immune responses, vascularization, matrix remodeling, and long-term tissue integration. Future development therefore requires clearly defined critical quality attributes and mechanism-linked potency assays for the final product rather than the isolated characterization of individual components.

Preclinical evaluation must also become more predictive of clinical conditions. Many studies rely on young, healthy animals and short observation periods that do not reproduce aging, diabetes, radiation injury, chronic inflammation, or scar-prone tissue environments. Validation should progress toward clinically scaled defects, disease-relevant models, and longer follow-up. Outcomes should reflect tissue-specific function rather than morphological improvement alone. Outcomes should reflect the clinical objective of the intended indication rather than morphological improvement alone. For reconstructive applications, priority endpoints include durable defect repair, barrier restoration, vascular integration, mechanical function, sensory recovery, scar quality, and host-tissue integration. For regenerative-aesthetic applications, additional priorities include skin texture, elasticity, pigmentation, soft-tissue pliability, contour stability, durability of visible improvement, and patient-reported satisfaction.

Because safety liabilities differ fundamentally among living-cell products, extracellular vesicles, implantable biomaterials, injectable biostimulators, and nanomaterials, the safety assessment should remain platform-specific rather than being reduced to a single generic risk framework. Across these platforms, evaluation should extend beyond acute biocompatibility to delayed and cumulative risks, including tumorigenicity or ectopic differentiation, immune activation and fibrosis, infection or vascular complications, biodistribution and material persistence, and adverse effects associated with repeated treatment.

Outcome assessment should therefore follow a multidimensional framework rather than rely on a single surrogate endpoint. Structural outcomes should evaluate tissue architecture, defect coverage, thickness, volume, contour, and host integration; biological outcomes should assess vascularization, innervation, extracellular-matrix organization, inflammatory responses, and tissue-specific maturation. Functional outcomes should reflect barrier function, mechanical performance, sensory or motor recovery, perfusion, or other indication-specific functions. Aesthetic outcomes should include scar quality, pigmentation, texture, elasticity, pliability, symmetry, and contour stability, while patient-reported outcomes should capture satisfaction, perceived appearance, symptoms, and treatment burden. Safety outcomes should be evaluated separately and include infection, fibrosis, nodules or granulomas, vascular complications, ectopic tissue formation, and delayed or repeat-treatment adverse events. Durability should be incorporated across these domains through clinically relevant longitudinal follow-up.

Manufacturing and regulation remain closely connected to biological variability. Cell-, vesicle-, gene-, and combination products require standardized sourcing, sterility control, storage conditions, release criteria, and batch comparability. Highly personalized constructs may offer anatomical or biological precision but are difficult to scale, whereas off-the-shelf products require sufficient consistency across heterogeneous patients and treatment sites. Regulatory classification becomes particularly complex when a scaffold simultaneously functions as a device, drug-delivery platform, and biological product. Cost, treatment logistics, and ease of clinical use must therefore be considered during early-stage design rather than after efficacy has been established.

The next phase of the field should prioritize controlled regeneration over technological complexity. Advanced biofabrication, organoids, immune-responsive materials, cell-free therapeutics, nanotechnology, and artificial intelligence will be most valuable when they improve reproducibility, mechanistic precision, and patient-relevant outcomes. For regenerative aesthetics, this also requires a stronger distinction between transient cosmetic improvement and durable biological remodeling. Progress will depend on integrating material design, manufacturing, biological validation, and clinical assessment into a unified translational framework. Under this framework, regenerative technologies should be judged not by how sophisticated they appear experimentally, but by whether they can safely and consistently restore tissue structure, function, and long-term homeostasis.

## 14. Conclusions

Tissue engineering is shifting from passive tissue replacement toward instructive systems that coordinate cells, extracellular matrix, immune responses, vascularization, and molecular signaling. Biomaterials, biofabrication, cell-based and cell-free therapies, organoids, nanotechnology, and digital technologies are increasingly being developed as integrated regenerative platforms, but they occupy markedly different stages of translation. Selected biomaterials and aesthetic injectable products already have established clinical applications, whereas many cell-based constructs, extracellular-vesicle therapeutics, living bioprinted tissues, and organoid-based interventions remain predominantly preclinical or at an early translational stage. Future progress should therefore be judged not only by biological novelty, but also by the level of supporting evidence, regulatory clarity, reproducibility, safety, and durable functional or aesthetic benefit. In plastic and reconstructive surgery, their value lies in improving wound and scar repair, vascularized soft-tissue reconstruction, craniofacial and peripheral nerve regeneration, host integration, and durable restoration of form and function. In regenerative aesthetics, the same principles extend beyond transient volume correction toward controlled dermal remodeling and reproducible improvement in tissue quality. Future progress should therefore be judged by reproducibility, safety, functional integration, aesthetic durability, and clinically meaningful patient outcomes rather than technological complexity alone.

## Figures and Tables

**Table 3:** Evidence level, translational maturity, and clinical status of major regenerative technologies relevant to reconstructive and aesthetic medicine.

Technology/Intervention	Predominant Evidence Stage	Translational Maturity	Clinical/Regulatory Status	Principal Concern in Interpretation or Safety	Key Validation Need	References
Conventional acellular scaffolds and dermal substitutes	Preclinical studies and substantial human clinical experience; evidence is product-specific	Established for selected indications	Selected products are regulator-authorized and commercially available; status varies by material and indication	Infection, inflammatory response, fibrosis, variable host integration	Long-term comparative effectiveness, durability, and standardized product-specific outcomes	[17,227]
Autologous adipose tissue-, SVF-, and cell-assisted approaches	Preclinical mechanistic studies plus heterogeneous human clinical studies	Clinical use established for selected autologous procedures, but evidence varies by processing method	Used clinically in some settings; regulatory requirements for cell processing vary by jurisdiction	Heterogeneous cell composition, processing variability, fat resorption, uncertain equivalence among SVF, expanded MSCs, microfat, and nanofat	Standardized preparation, dose definition, controlled comparative trials, long-term volume retention	[65,66,67,68]
Culture-expanded MSC products	Extensive in vitro and animal evidence; selected early human studies	Early clinical/investigational for most reconstructive and aesthetic indications	Product- and jurisdiction-specific; should not be generalized from one indication to another	Donor variability, senescence, phenotypic drift, immunogenicity, ectopic differentiation	Identity and potency assays, dose standardization, long-term safety, controlled clinical efficacy	[65,69,70,71]
iPSC-derived regenerative constructs	Predominantly in vitro and preclinical transplantation studies	Experimental	No routine reconstructive or aesthetic clinical use	Residual pluripotent cells, tumorigenicity, genomic instability, incomplete maturation	Genomic stability, purification, long-term tumorigenicity, large-animal validation, clinical-scale manufacturing	[77,78,79,80,81,82]
Living 3D-bioprinted tissues	Predominantly in vitro and preclinical animal studies; large-animal validation remains technology-dependent	Experimental/preclinical	No routine clinical use of complex living bioprinted replacement tissues	Insufficient vascularization, manufacturing variability, sterility, poor long-term maturation	Large-animal validation, GMP-compatible production, vascularization, mechanical and functional durability	[94,95,96,97,98,99]
Extracellular-vesicle/exosome therapeutics	Extensive in vitro and small-animal evidence; selected ex vivo and limited early human studies	Preclinical to early clinical	Commercial aesthetic offerings exist in some settings, but regulatory status varies; no FDA-approved exosome product in the United States	Product heterogeneity, contamination, undefined dose, biodistribution, unvalidated therapeutic claims	Standardized isolation and identity criteria, potency assays, dose definition, randomized clinical trials, repeat-dose safety	[108,109,110,111,112,124,125,228]
Skin organoids	Primarily in vitro; selected preclinical transplantation studies	Experimental	Research and translational-testing platform rather than routine therapy	Residual pluripotency, immature phenotype, limited vascular/immune integration	Reproducible maturation, vascularization, safety, clinically scalable manufacturing	[152,154,156,157,158]
Organ-on-a-chip systems	In vitro modeling and validation studies	Research/enabling technology	Not a therapeutic product	Model-to-human validity, incomplete systemic physiology	Correlation with clinical outcomes, reproducibility, standardized validation criteria	[164,166,167,168]
HA-based skin-quality injectables/skinboosters	Human clinical studies and substantial commercial experience for selected products; evidence is formulation-specific	Clinical for selected products and indications	Selected HA products have regulator-authorized aesthetic indications; e.g., SKINVIVE has FDA-authorized indications for skin smoothness/appearance in defined anatomical sites	Vascular compromise, infection, product heterogeneity; hydration/skin-quality effects may be confused with regeneration	Product-specific controlled trials, durability, objective skin-quality and ECM endpoints	[217,222]
CaHA and PLLA biostimulatory injectables	Human clinical and histological evidence, with established aesthetic use for selected products	Clinically established for specified aesthetic indications	Selected CaHA and PLLA dermal implants have FDA-authorized aesthetic uses; regulatory status remains product- and indication-specific	Nodules, granulomatous reactions, irregular fibrosis, technique-dependent outcomes	Quantitative remodeling endpoints, product-specific durability, standardized dosing and injection techniques	[138,211,217]
PCL-based biostimulatory injectables	Preclinical mechanistic evidence plus human clinical experience	Clinical/commercial in selected markets; maturity varies by jurisdiction	Commercial availability is jurisdiction-dependent and should not be equated with approval of a general regenerative claim	Nodules, inflammatory response, long material persistence, technique dependence	Comparative prospective studies, histological correlation, long-term safety and durability	[138,211,212]
PN/PDRN-based injectables	Mechanistic/preclinical studies and heterogeneous human studies	Early to intermediate clinical evidence, depending on formulation and indication	Commercially used in some jurisdictions; regulatory classification and indications vary	Formulation heterogeneity, protocol variability, uncertain equivalence among products	Larger controlled trials, standardized formulations, dose–response studies, objective long-term endpoints	[215,216]

Note: 3D, three-dimensional; CaHA, calcium hydroxyapatite; ECM, extracellular matrix; FDA, U.S. Food and Drug Administration; GMP, good manufacturing practice; HA, hyaluronic acid; iPSC, induced pluripotent stem cell; MSC, mesenchymal stem/stromal cell; PCL, polycaprolactone; PDRN, polydeoxyribonucleotide; PLLA, poly-L-lactic acid; PN, polynucleotide; SVF, stromal vascular fraction. SKINVIVE is a commercial product name rather than an abbreviation.

## Data Availability

No new data were created or analyzed in this study. Data sharing is not applicable to this article.

## Abbreviations

The following abbreviations are used in this manuscript:

3D	Three-dimensional
ADSC	Adipose-derived stem cell
AI	Artificial intelligence
ASC	Adipose-derived stromal cell
BMP	Bone morphogenetic protein
CaHA	Calcium hydroxyapatite
CD	Cluster of differentiation
dECM	Decellularized extracellular matrix
DLP	Digital light processing
DPSC	Dental pulp stem cell
ECM	Extracellular matrix
EV	Extracellular vesicle
FGF	Fibroblast growth factor
GelMA	Gelatin methacryloyl
HA	Hyaluronic acid
HAp	Hydroxyapatite
hiPSC	Human-induced pluripotent stem cell
IL	Interleukin
iPSC	Induced pluripotent stem cell
MSC	Mesenchymal stem/stromal cell
PCL	Polycaprolactone
PDRN	Polydeoxyribonucleotide
PDLSC	Periodontal ligament stem cell
PLGA	Poly(lactic-co-glycolic acid)
PLLA	Poly-L-lactic acid
PN	Polynucleotide
SLA	Stereolithography
TGF-β	Transforming growth factor-beta
TNF-α	Tumor necrosis factor-alpha
α-SMA	Alpha-smooth muscle actin
β-TCP	Beta-tricalcium phosphate
